# Pediatric Fibrinogen PART I—Pitfalls in Fibrinogen Evaluation and Use of Fibrinogen Replacement Products in Children

**DOI:** 10.3389/fped.2021.617500

**Published:** 2021-04-21

**Authors:** Elise J. Huisman, Gemma Louise Crighton

**Affiliations:** ^1^Department of Hematology, Erasmus Medical Center (MC)—Sophia Children's Hospital, Rotterdam, Netherlands; ^2^Department of Clinical Chemistry and Blood Transfusion, Erasmus Medical Center (MC), Rotterdam, Netherlands; ^3^Department of Transfusion Medicine, Sanquin Blood Supply, Amsterdam, Netherlands; ^4^Department of Hematology, Royal Children's Hospital, Melbourne, VIC, Australia

**Keywords:** children, intensive care, fibrinogen, hypofibrinogenemia, Clauss, viscoelastic testing, cryoprecipitate, fibrinogen concentrate

## Abstract

Fibrinogen is a key coagulation protein, playing a critical role in hemostasis. It is the first factor to decrease to critical levels during bleeding. Hypofibrinogenemia is an important risk factor for bleeding in clinical settings, including pediatric surgery. Yet, the optimal measurement of fibrinogen levels is subject to debate, as is the critical threshold for intervention. Fibrinogen replacement may be provided by cryoprecipitate and fibrinogen concentrate. Whilst both products contain fibrinogen, they are not equivalent, each has its own advantages and disadvantages, especially for pediatric use. Unfortunately, medical literature to support fibrinogen replacement in children is limited. In this article we review the current diagnostic tools to measure fibrinogen, with respect to their use in the pediatric critical care setting. Secondly, we evaluate the different fibrinogen replacement therapies, focusing on cryoprecipitate and fibrinogen concentrate and examine their individual product characteristics, associated risks and benefits, different dosing strategies and specific pitfalls for use in children. We summarize by highlighting current knowledge gaps and areas for future research.

## Introduction

Fibrinogen (Factor I) is a key coagulation protein and plays a critical role in all aspects of normal hemostasis: from platelet aggregation, to clot formation, and fibrinolysis (1, 2). During major surgical bleeding, fibrinogen is the first hemostatic factor to decrease to critical levels (3). Fibrinogen has been identified as an important risk factor for bleeding in children undergoing surgery (4–6).

As an acute phase reactant, fibrinogen plays a pivotal role in tissue repair and maintaining hemostasis during tissue injury and inflammation (7). Levels are often elevated in children in the context of fever, sepsis and critical illness (8–10). However, if hypofibrinogenemia is present in critically ill children with sepsis, it is associated with increased in-hospital mortality (8, 9).

See “Pediatric Fibrinogen PART II—Overview of Indications for Fibrinogen Use in Critically Ill Children”, for further information about the clinical indications for fibrinogen supplementation in children.

### Fibrinogen Evaluation and Specific Pitfalls in the Pediatric Setting

The gold-standard for fibrinogen quantification is the clot-based Clauss fibrinogen assay. The Clauss-assay is a functional test that measures the time taken for plasma to clot in a high concentration of thrombin, with comparison against a reference plasma calibration curve (11). Patient factors that can impact the accuracy of the test include the presence of heparin and fibrinogen-degradation products (12). Similarly photo-optical clot detection systems for measuring fibrinogen may be affected by lipemia or hyperbilirubinemia (11). Hydroxyethyl starch (HES), a plasma volume expander used in some pediatric perioperative settings (13), can impair fibrin polymerization (13, 14) causing falsely reduced fibrinogen levels (6, 14). Additionally, direct thrombin inhibitors such as bivalirudin and argatroban can inhibit the thrombin used in the Clauss-assay causing falsely reduced levels (15).

Fibrinogen may also be evaluated by viscoelastic testing. The two most commonly used and evaluated in clinical trials in children are thromboelastography (TEG®) and thromboelastometry (ROTEM®). A qualitative indication of fibrinogen is obtained by inhibiting the platelet contribution to clot formation using cytochalasin D for ROTEM-FIBTEM and abciximab for TEG functional fibrinogen (TEG-FF) assay (16).

Whilst both TEG and ROTEM are based on similar methodologies, there are significant differences in operating characteristics, activators used, reporting nomenclature, and reference ranges. Parameters reported by each of the devices are not equivalent or interchangeable (16). One such example, is the measurement of hyperfibrinolysis, the TEG lysis index is measured 30 min after the maximum amplitude (MA), whereas the ROTEM lysis index is measured 30 min after the clotting time (17).

Viscoelastic measures of fibrinogen have benefits over the Clauss assay, since they are point-of-care test with quick turnaround times (16). Since they are performed using whole blood, they provide a global and visual hemostatic evaluation of hemostasis, from clot initiation and kinetics, through to clot degradation and fibrinolysis.

In the pediatric setting the usefulness and uptake of viscoelastic testing remains limited. There is a lack of standardization, with a scarcity of age-dependent reference ranges for the different reagents used with each viscoelastic method (18, 19). Studies are needed to define clear thresholds and targets for treatment in neonates and children (17). The maximum clot strength thresholds (Maximum Clot Firmness [MCF] for ROTEM or MA for TEG) that indicate the need for fibrinogen supplementation are debated. In addition, validated treatment algorithms using viscoelastic testing and fibrinogen supplementation are needed for the different neonatal and pediatric contexts (17).

Viscoelastic testing in infants and neonates may also be limited by the blood sample requirements; ROTEM requires at least 1.8 mL of whole blood and TEG 2.7 mL (20) although newer viscoelastic devices have smaller minimum sample requirements (17). Another limitation is that viscoelastic tests are unable to detect congenital bleeding disorders, such as platelet function disorders or von Willebrand disease (17).

Other techniques for measuring fibrinogen include Prothrombin-time (PT) derived tests, immunohematological assays (11), the dry-hematology method (21), and thrombin generation assays. These tests are not widely used or only available in research settings (22).

The fibrinogen level obtained will vary depending on the fibrinogen assay used (23). In adults, fibrinogen levels using the Clauss assay are typically reported between 150 and 450 mg/dL, but physiological variation is commonly seen. In children, age-related reference ranges are required for fibrinogen quantification, because of the age-dependency (the so called developmental hemostasis) in hemostatic proteins (24). The lowest fibrinogen levels are reported in fetuses and preterm neonates (25–27), and infants (28, 29). Similarly, there are age-dependent differences seen in viscoelastic testing results (19, 30–32). Pediatric age-specific reference ranges have been described for ROTEM parameters across pediatric age groups, including FIBTEM (31, 33) and for the TEG (19, 34).

Children also have qualitative differences in both fibrinogen and fibrinolysis (35). Neonates have a “fetal” form of fibrinogen (35), an altered fibrin network and clot structure (36), with an overall reduction in fibrinolytic activity (37), and often physiologically elevated D-dimers in the first 72 h of life (38).

The sensitivity of each fibrinogen assay refers to the lowest detectable and quantifiable amount of fibrinogen. The lowest reportable fibrinogen level will vary between individual laboratories but will be reported as less than a certain threshold, e.g., <50 mg/dL. In neonates, this can be rather imprecise, because physiological values can be this low.

*In summary, fibrinogen reference ranges vary depending on the test, the analyzer and reagents used, in addition to the age of the child, therefore it is important that local, assay-specific and age-specific reference ranges are developed. Utilization of adult reference intervals, particularly in the neonates has the potential to lead to overtreatment. A normal physiological (low) fibrinogen level in a non-bleeding, preterm neonate, for example, does not need correction*.

## Fibrinogen Supplementation

Cryoprecipitate and fibrinogen concentrate both effectively restore fibrinogen levels (39, 40), and are used in children to provide fibrinogen replacement during active bleeding or as prophylaxis to prevent bleeding (41, 42). Internationally there is variability in practice regarding the favored fibrinogen replacement product, reflecting local legislation, licensing and product availability.

### Cryoprecipitate

In the United States (US), Canada, United Kingdom (UK), Australia and New Zealand, cryoprecipitate is the main component available for treatment of acquired hypofibrinogenemia since fibrinogen concentrate is only licensed for the treatment of congenital fibrinogen deficiency (43).

### Fibrinogen Concentrate

In contrast, many European countries favor fibrinogen concentrate over cryoprecipitate for all therapeutic uses due to its superior pathogen safety profile and cryoprecipitate has been withdrawn due to safety concerns, principally transfusion transmitted infection (TTI) and prions. Fibrinogen concentrate in Europe is licensed for treatment of both congenital and acquired hypofibrinogenemia (43).

Whilst cryoprecipitate and fibrinogen concentrate are both plasma-derived products, there are considerable differences between the two products (see Table 1).

**Table 1:** Comparison of Cryoprecipitate and Fibrinogen Concentrate (RiaSTAP ®/Haemocomplettan ® CSL Behring).

	**Cryoprecipitate (1–8)**	**Fibrinogen concentrate (RiaSTAP^®^/Haemocomplettan^®^) (8–11)**
**Plasma source**	Apheresis single donor or pooled from multiple donors Many countries only use male plasma donors to reduce the risk of TRALI.	Pooled from >10,000 plasma donors.
**Content**	Fibrinogen, vWF, FVIII, FXIII Platelet microparticles Fibronectin Anticoagulant—e.g., citrate-phosphate-dextrose or ACD	Fibrinogen Albumin L-arginine HCL
**Presentation**	Yellow, frozen cold-insoluble precipitate	White lyophilized powder
**Storage**	−25°C of below for a maximum of 3 years	2–6°C for up to 5 years
**Compatibility**	ABO compatibility with recipient's red cells suggested	No compatibility requirements
**Reconstitution**	Thawed at 37°C	Sterile water for reconstitution at room temperature
**Speed of preparation**	17–20 min to thaw	5–10 min
**Administration**	Standard blood administration set with a 170–200 micron filter.	Direct intravenous infusion by separate infusion line
**Shelf life**	Used within 4 h after thawing at room temperature	Stable for 8 h after reconstitution when stored at room temperature
**Fibrinogen content**	Variable fibrinogen content depending on fibrinogen content and cryoprecipitate volume Range from 300 to 3,000 mg/dL	Standardized fibrinogen content—1,000 mg/vial and 2,000 mg/vial
**Pediatric infusion**	10–20 ml/kg/h	Slow IV infusion, not exceeding 100 mg/min
**Pathogen-reduction and viral inactivation procedures**	Pathogen- reduced plasma—Photoactivation by visible or UV light, solvent-detergent treatment, or the addition of chemicals to the plasma: methylene blue (MB), amotosalen or riboflavin. Results in 65–84% lower fibrinogen content.	Al(OH)_3_ adsorption/glycine precipitation /Al(OH)_3_ adsorption Heat treatment at 60°C for 20 h Glycine precipitation
**Adverse events**	Risk of transfusion-transmitted infection Allergic transfusion reaction and anaphylaxis Febrile non-hemolytic transfusion reactions TRALI Citrate-induced hypocalcemia (increased risk with massive transfusion)	Allergic reactions and anaphylaxis Infusion related adverse events Thromboembolic complications
**Surveillance**	Hemovigilance	Pharmacovigilance
**Costs per gram fibrinogen**	~AUD $480 per gram Higher costs when cryoprecipitate is produced from pathogen-reduced plasma.	~AUD $817 per gram

*ACD, acid-citrate-dextrose; FVIII, factor VIII; FXIII, factor XIII; HCL, hydrochloride; IV, intravenous; TRALI, transfusion-associated acute lung injury; UV, ultraviolet; vWF, von Willebrand factor*.

### Plasma Transfusion

Plasma is the liquid component of blood that contains coagulation factors and coagulation inhibitors. Plasma products available include plasma frozen within 6–8 h of collection (FFP), plasma frozen within 24 h of collection, thawed plasma, liquid plasma and pathogen-inactivated plasma (44–46). Freeze-dried (lyophilized) or spray-dried plasma is largely restricted to military and research settings (47).

In the pediatric setting however, none of these plasma products, are suitable as fibrinogen replacement products since fibrinogen concentrations in plasma are very low and can vary considerably (100–500 mg/dL) (48, 49). Large volumes of plasma are required to replenish a low or falling fibrinogen level (43), placing a child at significant risk of transfusion-associated circulatory overload (TACO).

*In summary, cryoprecipitate and fibrinogen concentrate are superior to plasma as fibrinogen replacement products since they are concentrated products. We therefore will focus this review on these two fibrinogen products*.

## Product Characteristics

### Product Characteristics of Cryoprecipitate

Cryoprecipitate is the unpurified, cold-insoluble protein or “cryoglobulin” proportion derived from plasma. Cryoprecipitate may be manufactured as single apheresis units or pooled from multiple donors with varying volume size. It is stored as a frozen product and inventoried by blood group. It contains fibrinogen in addition to coagulation factors VIII (FVIII), von Willebrand factor (vWF), factor XIII (FXIII), as well as fibronectin and platelet microparticles (45). Cryoprecipitate was originally used therapeutically in the treatment of hemophilia A, and then von Willebrand disease (vWD), congenital fibrinogen and FXIII deficiency (50). Today cryoprecipitate is predominantly utilized to replace fibrinogen in acquired hypofibrinogenemia.

Internationally, specific product requirements for the manufacture of cryoprecipitate vary between countries with respect to the minimum fibrinogen, FVIII and vWF concentrations (44) (see Table 2). Whilst minimum fibrinogen content per unit is usually specified by standards (e.g., >140 mg/unit) (45, 46), the actual concentration can vary considerably (300–3,000 mg/dL), due to differences in blood donor fibrinogen levels, varying manufacturing processes and different unit volumes (51).

**Table 2:** International standard product specifications for cryoprecipitate.

	**Fibrinogen (mg) per unit**		**FVIII (IU) per unit**	**FXIII (IU) per unit**	**VWF per unit**	**Volume**
**United States** **(****1**, **2****)**	≥150^a^		≥80	≥80	≥80	Maximum 15 mL
**EDQM Standards 2020** **(****3****)**	≥140		≥70	Not specified	≥100	30–40 mL
**United Kingdom** **(****4**, **5****)**	Single unit	≥140^b^^,^ ^c^	≥70^b^	Not specified	Not specified	20–60 mL^d^
	Pool (5 donors)	≥700	≥350			100–250 mL^e^

a *average fibrinogen content 250 mg unit*,

b *a minimum of 75% of components tested should meet the specified values*,

c *NHBTS mean 454 mg*,

d *NHBTS mean 40 mL (4)*,

e *NHBTS mean 221 ml*.

Advantages and disadvantages of cryoprecipitate in comparison with fibrinogen concentrate are described in Table 1.

One potential clinical advantage of cryoprecipitate is the contribution of FXIII in the treatment of bleeding, especially in the surgical setting (52–54). *In-vitro* studies have shown that cryoprecipitate reverses fibrinolysis better than fibrinogen concentrate (55). A second consideration is cost: when evaluated per gram of fibrinogen, cryoprecipitate is cheaper (56, 57), even when economic analysis has considered preparation and wastage costs (58). However, this advantage may not be as notable with pathogen-reduced cryoprecipitate, since pathogen-inactivation comes with additional costs.

An important downside of cryoprecipitate is the risk of pathogen transmission, even with appropriate blood donor and donation screening (59). Cryoprecipitate made from pathogen-reduced plasma can reduce this risk (60–62) and this is the preferred product for the treatment of congenital bleeding disorder in resource-limited settings (60, 61). Unfortunately, pathogen-inactivation results in reduced fibrinogen levels (65–84%) of normal plasma (62). To overcome this, it may be possible to increase the number of donors contributing to the plasma pool or increase the transfusion volume (63, 64).

Cryoprecipitate has been implicated in a number of transfusion-related adverse events including allergic reactions, febrile, non-hemolytic transfusion reactions (65, 66) as well as transfusion-associated acute lung injury (TRALI) (67, 68) and TTI (59, 69). There is also evidence that adverse transfusion reactions occur more frequently in children compared with adult (66, 70). Any patient receiving cryoprecipitate must be monitored for a transfusion reaction and any incidents should be reported to the local hemovigilance reporting system. To maintain traceability, the blood compatibility report should be maintained in the child's medical record.

Cryoprecipitate is less suited to the acute pediatric critical care setting since it requires thawing prior to administration, and once thawed, it has a shelf life of 4 h, after which it must be discarded (46). To overcome this, it would be ideal to keep “ready for-use” units of unthawed cryoprecipitate. *In-vitro* studies evaluating thawed pathogen-reduced cryoprecipitate, held at room temperature for 5 days have demonstrated its ability to restore fibrinogen levels and clot strength (FIBTEM) (63). However, in the pediatric setting di (2-ethylhexyl)phthalate (DEHP) toxicity should be considered (71). DEHP is a chemical added to plastics to make them more flexible and is commonly used in blood bags (72). DEHP levels increase in concentration as blood storage duration is increased (71). Toxic and carcinogenic effects are seen in animal studies, but the evidence for toxicity in humans has not been established, but remains a concern in neonates (72).

### Product Characteristics of Fibrinogen Concentrate

Fibrinogen concentrate is a purified, virus-inactivated, lyophilized concentrate derived from pooled human plasma. It comes as a powder that can be reconstituted with sterile water (within 5–10 min) to deliver a reliable and standardized fibrinogen content (73, 74). In children with hypofibrinogenemia, fibrinogen concentrate has a rapid onset of action and is effective at increasing fibrinogen levels (39, 40).

There are a number of fibrinogen concentrates available internationally. The one most widely used is Haemocomplettan P®/RiaSTAP® (CSL Behring) (43) which contains between 900 and 1,300 mg of fibrinogen/50 mL (74, 75). Other available fibrinogen concentrates include: Clottafact® (LFB Biomedicaments) (76), FibCLOT® (LFB Biopharmaceuticals Ltd.) (77) and Fibryga®/Octafibrin ® (Octapharma) (78) and locally produced Fibrinogen HT (Japan) and FibroRAAS/FabuLaishi (China) (79). These fibrinogen concentrates are not all equivalent, they differ in: pathogen reduction strategies (80), fibrinogen concentrations, formulations (for example Fibryga® Octapharma contains more FXIII) (81, 82), stability agents and constituents, storage requirements and reconstitution stability data, and finally, in varying pediatric dosing recommendations (43, 74, 76–78, 81, 83, 84) (see Table 3) for the full overview.

**Table 3:** Comparison between fibringeon concentrate products.

	**RiaSTAP^®^/Haemocomplettan P^®^ (1–3)**	**Fibryga^®^/Octafibrin^®^ (4, 5)**	**Clottafact^®^/FibCLOT^®^ (6–9)**
**Manufacturer**	CSL Behring, Germany	Octapharma, Switzerland	LFB Biomedicaments, France
**Pathogen reduction**	Al(OH)_3_ adsorption/glycine precipitation /Al(OH)_3_ adsorption Heat treatment at 60°C for 20 h Glycine precipitation	Al(OH)_3_ adsorption Solvent-detergent treatment Nanofiltration (20 nm)	Solvent-detergent treatment Nanofiltration (35 nm) Dry heat treatment at 80°C for 20 h
**Vial size**	1,000 and 2,000 mg	1,000 mg	1,500 mg
**Fibrinogen concentration**	900–1,300 mg/50 mL 20 mg/ml	1 g/50 mL 20 mg/mL	1.5 g/100 mL 15 mg/ml
**Stability agents**	Albumin L-arginine HCL Sodium chloride Sodium citrate	Glycine L-arginine HCL Sodium chloride Sodium citrate dihydrate	Glycine Arginine HCL Isoleucine Lysine HCL Sodium citrate dihydrate
**FXIII**	N/A	200IU FXIII/g of fibrinogen	N/A
**Storage**	2–5°C Up to 60 months	2–25°C Up to 36 months	3 years
**Reconstitution stability data**	Use within 8 h	Up to 24 h at +25°C	After reconstitution, use immediately
**Pediatric Dosing**	Dose (mg/kg body weight) = [Target level (mg/dL)—measured level (mg/dL)]/1.7 (mg/dL per mg/kg)	Dose (mg/kg body weight) = [Target level (mg/dL)—measured level (mg/dL)]/1.8 (mg/dL per mg/kg)	Dose (mg) = [Target level (mg/dL)—baseline level (mg/dL)] × 1/recovery (mg/dL)/(mg/kg) × body weight (kg)
**Pediatric dosing when fibrinogen level unknown**	RiaSTAP® 70 mg/kg for congenital fibrinogen deficiency	60 mg/kg for congenital fibrinogen deficiency	When 1/recovery unknown 53 mg/kg for children <40 kg 43 mg/kg for children ≥40 kg
	Haemocomplettan P® 20–30 mg/kg treatment for active bleeding in children		
**Pediatric infusion rates**	Slow IV infusion, not exceeding 100 mg/min	Slow IV infusion, maximum rate of 100 mg/min.	Clinically stable patients—4 ml/min = 60 mg/min Severe acute hemorrhage—20 ml/min = 300 mg/min

*HCL, hydrochloride; IV, intravenous*.

An overview of the advantages and disadvantages of fibrinogen concentrate compared with cryoprecipitate are described in detail in Table 1. An advantage of fibrinogen concentrate for use in neonates and critically ill children is its relatively small infusion volume. Doses of fibrinogen concentrate are going to be in the order of 1–3.5 mL/kg compared with 5–10 mL/kg for cryoprecipitate. Fibrinogen may be able to be administered quicker than cryoprecipitate, depending on where it is stored in the hospital, since it only requires refrigeration for storage.

Fibrinogen concentrate also has a superior pathogen safety profile and low rates of adverse events. A pharmaco-surveillance study of 27 years of Haemocomplettan P®/RiaSTAP® (CSL Behring) use with literature review, reported an excellent safety profile (85). Each fibrinogen concentrate administration be monitored for side effects and any reactions or adverse events be reported to the pharmacovigilance system. Each administration and the batch number must be recorded in the patient record to maintain traceability.

A relative disadvantage of use in neonates is that the dose required for treatment is much less than the smallest formulation of fibrinogen concentrate available (1,000 mg), leading to product wastage and higher costs. For example, a dose of 30 mg/kg fibrinogen concentrate to treat hypofibrinogenemia in a 5 kg infant only equals 150 mg.

1. *In summary, cryoprecipitate is a rich source of fibrinogen and includes other coagulation factors important for hemostasis. Cryoprecipitate is widely used in countries where fibrinogen concentrate is not licensed for use in acquired hypofibrinogenemia, and in resource-limited settings as therapy for congenital hypofibrinogenemia, although pathogen-inactivated cryoprecipitate is favored. Disadvantages are the large inter-unit variability of fibrinogen in cryoprecipitate, the increased risk of transfusion reactions and infectious transmission, in addition to the logistical and blood banking requirements*.
2. *Plasma-derived fibrinogen concentrates are able to rapidly and effectively restore and maintain serum fibrinogen concentrations. They are used for treatment of congenital and acquired hypofibrinogenemia. They have an excellent safety profile and are amenable to near-patient storage, which may reduce the time to administration. They come with a higher cost and generally do not contain additional hemostatic factors that may be important for general hemostasis*.

## Dosing of Fibrinogen Supplements

### Dosing of Cryoprecipitate

A standard treatment dose of cryoprecipitate in adults is 10 units or two pools (where one pool is made from five units), leading to an increase of 100 mg/dL fibrinogen (86). In children, dosing for both prophylactic and therapeutic cryoprecipitate indications should be calculated at least taking into account the child's weight (87). Most pediatric transfusion guidelines dose cryoprecipitate based on the child's weight as a single variable (88, 89). Many advise doses of 5 and 10 mL/kg (88–91), with exceptions of 20 mL/kg for the treatment of congenital fibrinogen deficiency with pathogen-reduced cryoprecipitate (64) (see Table 4).

**Table 4:** Guidance for dosing of cryoprecipitate and fibrinogen concentrate in children.

**Cryoprecipitate dosing in children**		
**Guidance**	**Dosing**	**Dosing considerations**
National Blood Authority of Australia (NBA) (2)	5 ml/kg for acquired hypofibrinogenemia	Consider patient's clinical condition, the presence of active bleeding, medications affecting coagulation status, and congenital and acquired bleeding disorders.
British Society of Hematology (BSH) (3)	5–10 ml/kg of methylene blue cryoprecipitate Higher doses for active bleeding and acquired hypofibrinogenemia	Lower fibrinogen content in methylene-blue cryoprecipitate ~250 mg/unit compared with ~430 mg/unit in a non-treated unit (4).
Italian Neonatal Transfusion Guideline, 2015 (5)	5–10 ml/kg	
Rare bleeding disorder guideline (64)	15–20 ml/kg of pathogen reduced cryoprecipitate for treatment of congenital fibrinogen disorders when fibrinogen concentrate not available	Fibrinogen concentrate is first line treatment for congenital fibrinogen disorders
Society for the Advancement of Blood Management (SABM) (7)	Cryoprecipitate volume should be calculated based on weight and desired increase in fibrinogen concentration and improvement in coagulations indices.	The decision to transfuse should be based on laboratory studies including point of care viscoelastic testing if available, fibrinogen concentration, the patient's clinical status and the etiology of the patient's coagulopathy.
US Food and Drug Administration (FDA) (8)	Dose of 0.1–0.2 units/kg 1–2 units per 10 kg	Unit volumes will vary. Expected fibrinogen rise of 60–100 mg/dL
AABB (9)	Calculated from formula $Dose\;\left(units\right)=\frac{\left[desired\;fibrinogen\;increment\;\left(\frac{mg}{dL}\right)x\;plasma\;volume\;\left(dL\right)\right]}{250mg}\;$ $Plasma\;volume\;\left(dL\right)\;in\;children=Total\;Blood\;volume\;\left(mL\right)\;x\frac{\left[1-hematocrit\;\left(\%\right)\right]}{100}\;$	Takes into account the desired fibrinogen increment (mg/dL), average fibrinogen content per unit of 250 mg and plasma volume of the child (9). Plasma and blood volumes vary in children. Blood volumes in children may be estimated using the formula 70 mL/kg, but this will underestimate the blood volume in a neonate and overestimate the blood volume in an adolescent or overweight child (10, 11)
Blood Easy 4, Canada, 2016 (12)	1 unit/10 kg body weight to a maximum of 10 units (~4,000 mg fibrinogen)	Each dose should increase the fibrinogen by 50 mg/dL in the bleeding patient.
NICE Guidelines 2015 (13)	5–10 mL/kg up to a maximum of two pools	Reassess the patient's clinical condition, repeat the fibrinogen level measurement, and give further doses if needed.
**Fibrinogen concentrate dosing in children**		
**Pediatric dosing**	**Dosing**	**Dosing considerations**
Riastap ® (CSL Behring, Germany) (14)	**Congenital fibrinogen deficiency**	70 mg/kg when patient's fibrinogen level is not known
	$Dose\;\left(\frac{mg}{kg}\right)=\;\left[Target\;fibrinogen\;level\;\left(\frac{mg}{dL}\right)\;-measured\;level\;\left(\frac{mg}{dL}\right)\right]$ / 1.7(*mg/dL per mg/kg body weight*)	
Rare bleeding disorder guideline (6)	**Congenital fibrinogen deficiency**	Smaller doses repeated every 2–4 days to maintain fibrinogen >100 mg/dL
	50–100 mg/kg	
Fibryga ® Octapharma	**Congenital fibrinogen deficiency**	60 mg/kg when patient's fibrinogen level is not known Monitor patient's fibrinogen level during treatment
	$Dose\;\left(\frac{mg}{kg}\right)=\;\left[Target\;fibrinogen\;level\;\left(\frac{mg}{dL}\right)\;-measured\;level\;\left(\frac{mg}{dL}\right)\right]$ / 1.8 (*mg/dL per mg/kg body weight*)	
Haemocomplettan P ® (CSL Behring, Germany) (15)	**Acquired hypofibrinogenemia**	
	20–30 mg/kg	

However, more complex formulas will take into account a child's baseline fibrinogen level, the desired fibrinogen level, the average fibrinogen content of the local cryoprecipitate unit and a child's plasma volume (92) (see Table 4).

In general, blood volumes in children may be estimated using the formula 70 mL/kg (93, 94). However, this equation underestimates the blood volume for a neonate, since the estimated blood volume (EBV) decreases with age, from around 90–100 mL/kg in preterm infants to ~80 mL/kg in term infants (93) and overestimates the blood volume in obese, post-pubertal adolescents (~60–70 mL/kg). None of these calculations corrects for ethnic differences and social factors such as malnutrition or obesity which may be important (94).

Dosing of cryoprecipitate in children should also consider the fibrinogen content of local products, particularly with pathogen-reduced cryoprecipitate (92, 95, 96).

The half-life of fibrinogen is relatively long (3–4 days) (64), and therefore, usually one dose is sufficient for prophylactic indications, however in the presence of active bleeding with ongoing loss or a poor fibrinogen increment, additional doses may be required.

### Dosing of Fibrinogen Concentrate

Fibrinogen concentrate is dosed by most clinical guidelines in mg/kg. But here also, more complex formulas are seen, taking target and measured fibrinogen levels into the equation (see Table 4).

For each of the other available fibrinogen concentrates on the market, there are different dosing recommendations for children, and it is therefore recommended to consult the individual product information for specific dosing advice (see Table 3).

*In summary, the dosing of cryoprecipitate is influenced by both donor and product variables. The dosing of both cryoprecipitate and fibrinogen concentrate in children should be calculated, taking into consideration the baseline fibrinogen, the child's body weight, the presence of active bleeding and ongoing loss. It is advisable, that after fibrinogen supplementation, both the clinical response to treatment and fibrinogen levels are re-evaluated to assess for any additional requirements*.

## Current Knowledge Gaps, Controversies, and Areas for Research

The decision to supplement fibrinogen firstly relies on adequate measurement of fibrinogen. When evaluating fibrinogen levels and function in the critically ill child, it is important to consider the accuracy of the result, the clinical context, the specific reference ranges and each test's limitations.

When fibrinogen replacement is indicated in critically ill children, there remain many uncertainties regarding the best choice of fibrinogen replacement, the optimal dose, in addition to the target or desired fibrinogen level. Each product has its own set of limitations and benefits specific to children.

The following questions, may be proposed as potential areas for research in neonates, including those preterm, infants, children, and adolescents.

In the area of laboratory testing:

- Can we develop small-volume tests for measuring fibrinogen in neonates and critically ill children?
- How do different viscoelastic measures of fibrinogen function compare with clot based Clauss fibrinogen assays in children?

Fibrinogen and bleeding

- How do age-dependent coagulation differences influence bleeding in neonates and children?
- In particular, what effect does the presence of fetal fibrinogen and altered fibrin clot structure have on fibrinogen function in preterm and term neonates?

Fibrinogen replacement products

- What is the optimal fibrinogen replacement component in children?
- What role does the additional FXIII, vWF, and FVIII in cryoprecipitate play in treating and preventing bleeding in children with a low fibrinogen?
- How do fibrinogen concentrate products that contain FXIII compare with those that do not?
- What is the optimal formula to dose fibrinogen in children?
- What is the optimal dose and timing in children of cryoprecipitate and fibrinogen concentrate
  ° for the prevention of bleeding?
  ° for the treatment of active bleeding?

Adverse events:

- How do we capture and report adverse events related to fibrinogen supplementation in children?

## Conclusions

Hypofibrinogenemia is increasingly recognized as an important risk factor for bleeding and there has been an increasing focus on the fibrinogen supplementation by clinicians and clinical guidelines. Yet, there are many unknowns. The decision to supplement fibrinogen firstly relies on adequate measurement of fibrinogen and there are many pitfalls around the optimal fibrinogen measurement in children. Cryoprecipitate and fibrinogen concentrate both effectively restore fibrinogen levels, but each product has its own set of advantages and constraints specific to use in children. Fibrinogen concentrate is an attractive alternative to cryoprecipitate, offering a superior safety profile, with apparent efficacy, but not every fibrinogen concentrate product is equivalent. Further randomized controlled evidence is required to support decision- making regarding fibrinogen supplementation in children, including those who are critically ill.

## Author Contributions

GC literature review, writing, and editing article. EH reviewing content and editing article. All authors contributed to the article and approved the submitted version.

## Conflict of Interest

The authors declare that the research was conducted in the absence of any commercial or financial relationships that could be construed as a potential conflict of interest.
